# Differential Susceptibility to Porcine Deltacoronavirus: Ducks Show Greater Vulnerability Than Geese

**DOI:** 10.1155/tbed/2339024

**Published:** 2025-06-04

**Authors:** Renqiang Liu, Shuhuai Meng, Lei Shuai, Hao Zhang, Guangli Hu, Huijuan Guo, Jianfei Chen, Dan Shan, Yakun Du, Yongchang Cao, Zhigao Bu, Zhiyuan Wen

**Affiliations:** ^1^State Key Laboratory for Animal Disease Control and Prevention, Harbin Veterinary Research Institute, Chinese Academy of Agricultural Sciences, Harbin 150069, Heilongjiang, China; ^2^State Key Laboratory of Biocontrol, School of Life Sciences, Sun Yat-sen University, Guangzhou 510006, Guangdong, China; ^3^Jiangsu Co-innovation Center for Prevention and Control of Important Animal Infectious Diseases and Zoonoses, Yangzhou University, Yangzhou 225009, Jiangsu, China

## Abstract

Porcine deltacoronavirus (PDCoV), an enteropathogenic coronavirus belonging to the *Deltacoronavirus* genus, is globally distributed and represents a significant viral enteric disease in pigs. It primarily causes severe diarrhea in pigs, especially in newborn piglets, with high fatality rates, resulting in substantial losses to the pig farming industry. Notably, PDCoV is also recognized as a zoonotic virus. PDCoV is a coronavirus that can infect both birds and mammals, including humans, thereby potentiating its zoonotic prevalence. However, the PDCoV circulation among birds and mammals remains poorly understood. Considering the close relationship and large farming numbers of domestic waterfowl, they may play crucial roles in PDCoV interspecies transmission, albeit their susceptibility remains unknown. In this study, we investigated the susceptibility of specific pathogen free (SPF) Shaoxing ducks and clean (CL) animal Chinese white geese to PDCoV. These results indicated that Shaoxing ducks and Chinese white geese are susceptible to PDCoV infection, viral RNA can be detected in intestines, parenchymal organs, and feces, though the clinical signs of diarrhea in ducks are significantly more severe than in geese. Histopathological analysis revealed obvious lesions in the intestines of infected Shaoxing ducks, while no lesions were observed in Chinese white geese. Moreover, the infected ducks exhibited diarrhea, and PDCoV seroconversion occurred 14 days postinoculation (dpi). Thus, our study broadens the spectrum of PDCoV hosts and provides valuable information for further investigation of PDCoV interspecies transmission. Waterfowl, especially ducks, may play an important role in the inter-species transmission of the PDCoV.

## 1. Introduction

Coronaviruses are enveloped, single-stranded, and positive-sense RNA viruses belonging to the *Coronaviridae* family, order *Nidovirales* [1]. Coronaviruses are categorized into four genera: *Alphacoronavirus*, *Betacoronavirus*, *Gammacoronavirus*, and *Deltacoronavirus* [2]. *Deltacoronaviruses* mainly infect birds and it was initially detected in wild Asian leopard cats and Chinese ferret badgers in southern China [3]. Subsequently, a novel avian deltacoronavirus was discovered during molecular surveillance in birds and mammals in Hong Kong from 2007 to 2012. Porcine deltacoronavirus (PDCoV) was also first identified during this period [4, 5]. PDCoV causes severe watery diarrhea and intestinal lesions in piglets. The first reported outbreak of PDCoV in swine populations occurred in Ohio, USA, in 2014 [6]. Since then, successive outbreaks in several countries, including Canada [7], China [8], South Korea [9], Thailand, Laos, and Vietnam [10], have resulted in significant economic losses for the swine industry. Recently, PDCoV has been isolated from the plasma samples of three Haitian children with acute febrile illnesses [11], which reveals that PDCoV has the ability to cross species barriers and poses a potential threat to human health.

The origin and transmission pathways of PDCoV are currently unknown. Previous studies have suggested that the transmission of the deltacoronavirus from birds to pigs has led to the emergence of PDCoV [12]. In 2017, avian deltacoronaviruses were detected in wild sparrow feces collected from swine farms experiencing PDCoV outbreaks in the United States [13]. Additionally, the *S* gene of PDCoV has high sequence similarities with deltacoronaviruses isolated from Asian leopard cats and Chinese ferret badgers, with a nucleotide similarity exceeding 99.8% [5]. It is, thus, speculated that the emergence of PDCoV is associated with the interspecies transmission of deltacoronavirus among birds and several mammalian species and the full extent of its interspecies transmission potential remains unclear.

Experimental infections in various animals confirm the broad host range of PDCoV. Chicken embryos and specific pathogen free (SPF) chickens and farm turkeys exhibit susceptibility to PDCoV [14, 15]. Among mammals, calves only show mild clinical signs with PDCoV infection [16], while mice are also susceptible. The reported detection of PDCoV in human blood indicates the potential threat to human health, which highlights the necessity to clarify its interspecies transmission. However, the transmission chain remains obscure, emphasizing the importance of conducting susceptibility studies in animals. Ducks and geese, being widely cultivated domestic waterfowl across the globe, play a pivotal role in livestock production and often reside near human settlements. Therefore, it is imperative to understand the susceptibility of these waterfowl to PDCoV and to assess their significance in the transmission and evolution of the virus. To the best of our knowledge, the infection of PDCoV in domestic waterfowl has not been reported. The current study confirms that both ducks and geese can be infected by PDCoV and manifest clinical signs and tissue pathological damages, thus, indicating the role of domestic waterfowl in the interspecies transmission of PDCoV. These findings widen the known host range of PDCoV infection and suggest that domestic waterfowl may play a significant role in the interspecies transmission of PDCoV.

## 2. Materials and Methods

### 2.1. Virus and Cells

PDCoV CHN-GD-2016 strain (GenBank: MF280390.1) was used [17]. Virus was passaged in LLC-PK cells (porcine proximal tubular cells). These cells were cultured in Dulbecco's Modified Eagle Medium (DMEM; Sigma–Aldrich, USA) supplemented with 10% fetal bovine serum (ExCell, China). Virus titers were determined using the tissue culture infectious dose 50 (TCID_50_) assay in LLC-PK cells.

### 2.2. Animals

SPF Shaoxing ducks and clean (CL) animal Chinese white geese (*Anser cygnoides domesticus*) were obtained from China National Experimental Poultry Resource Repository. According to the Chinese National Standard GB 14922.2-2011, the definition of CL animal is as follows [18]: In addition to the pathogens excluded in conventional animals, these animals are further free from pathogens that pose significant risks to animal health or substantially interfere with scientific research outcomes. Their production and testing complied with the requirements of the Pharmacopoeia of the People's Republic of China (2015 edition) national standards. All animals were housed in isolation at the Animal Experimental Center of Harbin Veterinary Research Institute, Chinese Academy of Agricultural Sciences, and were provided with sterile feed and water. The experimental protocol and facility setup were approved by the Animal Ethics Committee of the Harbin Veterinary Research Institute, Chinese Academy of Agricultural Sciences (Approval No. 2303110-02-GR).

### 2.3. Virus Inoculation

Four-day-old ducks or geese (*n* = 40) were randomly divided into two groups: the inoculated group (*n* = 25) and the control group (*n* = 15), which were housed in separate isolation units. Each animal in the inoculated group was orally inoculated with 1 × 10^6^ TCID_50_ of PDCoV in 300 μL of DMEM, while the control group received the same volume of DMEM only as mock infection. Daily observations and recordings of diarrhea scores began on 1 day postinoculation (dpi). The fecal scoring system included four levels: 0 = normal feces, 1 = soft but formed feces; 2 = semiliquid feces; 3 = watery diarrhea; with a score of 2 or above considered as diarrhea.

### 2.4. Sample Collection

Fecal samples were collected daily using cotton swabs from 1 dpi. All swabs collected with fecal samples were suspended in 1 mL DMEM and centrifuged at 1800 × *g* for 5 min at 4°C. The supernatant was then used for viral RNA extraction. On 3, 5, 7, 10, and 14 dpi, five animals from the inoculation group and three animals from the control group were euthanized under deep anesthesia and organ samples were collected for virus distribution detection, including heart, liver, spleen, lungs, kidneys, duodenum, pancreas, jejunum, ileum, cecum, and rectum. Euthanasia was performed by introducing 75% carbon dioxide into the isolator housing the animals. After confirming the absence of reflexes and loss of consciousness, cervical dislocation was carried out to ensure death, followed by organ sample collection. The euthanasia procedure was conducted in accordance with the AVMA Guidelines for the Euthanasia of Animals: 2020 Edition. Fresh samples were used for qRT-PCR and reverse transcription (RT)-PCR, and remaining samples were fixed in formalin for pathological examination. On 14 dpi, blood was collected from five animals in the inoculation group and three animals in the control group for antibody detection.

### 2.5. Virus RNA Extraction, qRT-PCR, and RT-PCR

Approximately 100 mg fresh organ samples were immersed in 1 mL phosphate-buffered saline (PBS) and then homogenized into a suspension using a FastPrep-24 5G homogenizer (MP Biomedicals, USA). The homogenized samples were centrifuged at 2000 × *g* for 10 min at 4°C to remove debris. Viral RNA was extracted from 300 μL of the supernatant using the Virus DNA/RNA Extraction Kit 2.0 (Vazyme, China) according to the manufacturer's instructions. RT was performed using the HiScript III 1st Strand cDNA Synthesis Kit (Vazyme, China) to obtain total complementary DNA (cDNA). Using cDNA samples, qRT-PCR and RT-PCR were conducted to respectively detect the dynamic distribution of the virus within organs and the organ tropism of the virus. qRT-PCR primers and probe targeting the PDCoV *M* gene (FPD-M: ATCGACCACATGGCTCCAA, RPD-M: CAGCTCTTGCCCATGTAGCTT, and PROBE-M: FAM-CACACCAGTCGTTAAGCATGGCAAGCT-BHQ) were used to amplify a 72-bp fragment. RT-PCR primers targeting the PDCoV *S* gene (SF: CAACCGTCTTGAGGAAGTAGAG and SR: TCAACGGTGAGGTTGAGAATAG) were used to amplify a 609-bp fragment, all primers and probes were designed based on the genome sequence of CHN-GD-2016 strain (GenBank: MF280390.1). qRT-PCR was performed on a QuantStudio 5 fluorescence quantitative PCR instrument (Applied Biosystems, USA) and results were analyzed using built-in software. The detection limit of qRT-PCR was 4.6 log_10_ copies/g in organ and fecal samples. For each qRT-PCR reaction, PBS and samples of the control group were used as negative controls.

### 2.6. Histopathology

The organ samples of ducks and geese were collected and fixed with 4% paraformaldehyde (PFA) at room temperature for 7 days, including hearts, livers, spleens, lungs, kidneys, pancreas, jejunum, ileum, cecum, and rectum. Samples were then embedded, sectioned, stained with hematoxylin and eosin (H&E), and examined under an optical microscope.

### 2.7. Enzyme-Linked Immunosorbent Assay (ELISA) for Detection of PDCoV-Specific Antibodies in Serum

Indirect ELISA was employed to detect specific IgG antibodies against PDCoV in serum. In a 96-well plate, 5 µg inactivated PDCoV was coated at 4°C overnight, followed by blocking with 5% skim milk at 37°C for 2 h. Subsequently, 10 µL of duck and goose serum was serially diluted twofold in skim milk and the plate was incubated at 37°C for 1 h. Afterward, goat anti-duck IgG antibody (H + L; Kirkegaard and Perry Laboratories, USA) conjugated with horseradish peroxidase was added at a dilution of 1:250, followed by incubation at 37°C for 1 h. The plate was washed three times with PBST (PBS containing 0.05% Tween 20), incubated with 3,3′,5,5′-tetramethylbenzidine (TMB) for 15 min at 37°C and the reaction was stopped by adding sulfuric acid. Absorbance was read at 450 nm with 630 nm as the reference wavelength.

### 2.8. Sequencing of Duck/Goose PDCoV *S* Gene

The complete *S* gene of PDCoV from duck and goose samples was amplified, cloned, and sequenced. Primers PDS-F and PDS-R (sequence: PDS-F: ATGCAGAGAGCTCTAT and PDS-R: CTACCATTCCTTAAACTTA) were used to amplify the *S* gene, with an expected size of 3480 bp. Total RNA was extracted from organ samples as described above and cDNA was reverse transcribed using the HiScript III 1st Strand cDNA Synthesis Kit (Vazyme, China). The *S* gene was amplified using KOD One PCR Master Mix DNA polymerase (TOYOBO, Japan) with the following PCR procedure: 98°C for 5 min (initial denaturation); 40 cycles of 98°C for 20 s (denaturation), 55°C for 15 s (annealing), and 68°C for 4 min (extension); followed by a final extension at 68°C for 10 min.

### 2.9. Statistical Analysis

*p* values for statistical analysis were determined using GraphPad Prism 8.0's one-way ANOVA function. The data are displayed as means ± standard errors of the means (SEM).

## 3. Results

### 3.1. Inoculated Animals Exhibit Diarrhea and Shed Viruses From the Intestine

**Figure 1:**
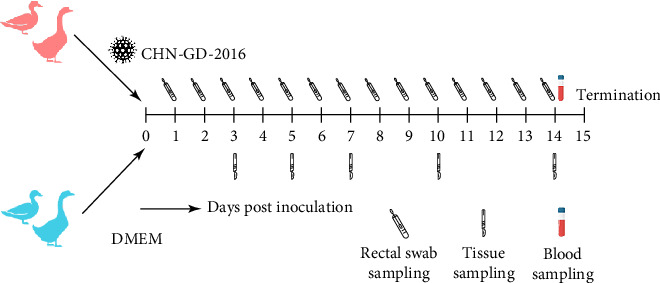
Experimental procedure. 1 × 10^6^ TCID_50_ PDCoV CHN-GD-2016 were orally inoculated to all animals in inoculated group. The ducks and geese are 4 days old and both received a 300 μL oral inoculation.

**Figure 2:**
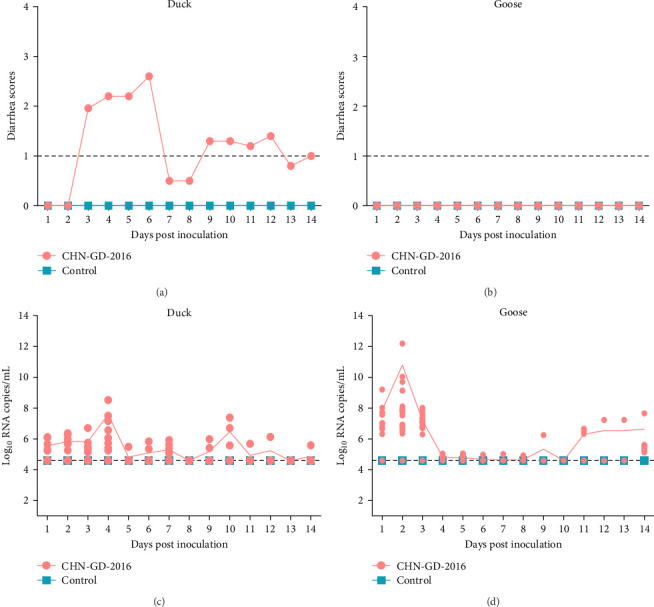
Diarrhea Scores (A, B) and fecal virus RNA shedding (C, D) in specific pathogen free (SPF) ducks and clean (CL) animal geese inoculated with porcine deltacoronavirus (PDCoV) strain CHN-GD-2016. Diarrhea scores for ducks (A) and geese (B) were monitored daily postinoculation. Viral RNA shedding in ducks (C) and geese (D) feces was determined using quantitative real-time RT-PCR (qRT-PCR). The qRT-PCR detection limit for virus RNA in fecal samples was 4.6 log_10_ copies/mL of PDCoV as shown by the dotted lines in the figure.

To assess the susceptibility of ducks and geese to PDCoV, 4-day-old SPF ducks and CL geese were orally inoculated with the CHN-GD-2016 strain. The observations of diarrhea and collection of cloacal swab samples were performed daily until 14 dpi (Figure 1). Ducks showed evident diarrhea at 3 dpi (Figure 2A), with fecal viral RNA peak levels at 2 dpi (around 11 Log_10_ RNA copies/mL; Figure 2C). Geese did not exhibit obvious diarrhea throughout the experimental period (Figure 2B) and fecal viral RNA levels increased twice at 4 dpi (around 8.00 Log_10_ RNA copies/mL) and 10 dpi (around 6.5 Log_10_ RNA copies/mL; Figure 2D). Animals in the control group showed no diarrhea. Viral RNA was not detected in fecal samples.

### 3.2. Viral Genomes Detected in the Intestine and Other Parenchymal Organs of Inoculated Animals

**Figure 3:**
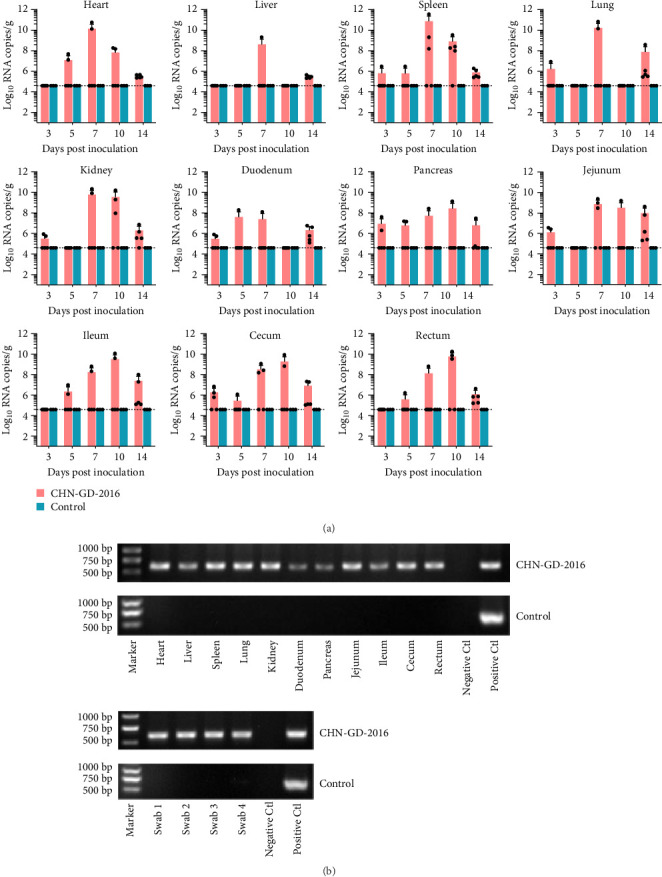
Distribution of virus RNA in heart, liver, spleen, lungs, kidneys, duodenum, pancreas, jejunum, ileum, cecum, and rectum of ducks using qRT-PCR (A) and RT-PCR (B). The distribution and dynamics of PDCoV across different organs of inoculated and control ducks were evaluated through qRT-PCR at 3, 5, 7, 10, and 14 dpi (A). The specific amplification of the PDCoV *S* gene fragment in tissue samples from inoculated group were tested positive by qRT-PCR at 7 dpi via RT-PCR (B). For rectal swab, swab1-4 were random selected from samples which were tested positive by qRT-PCR from 2, 3, 11 and 14 dpi, respectively. The detection limit for viral RNA in organ samples by qRT-PCR was determined to be 4.6 log_10_ copies/g of PDCoV, indicated by the dotted lines in the figure.

**Figure 4:**
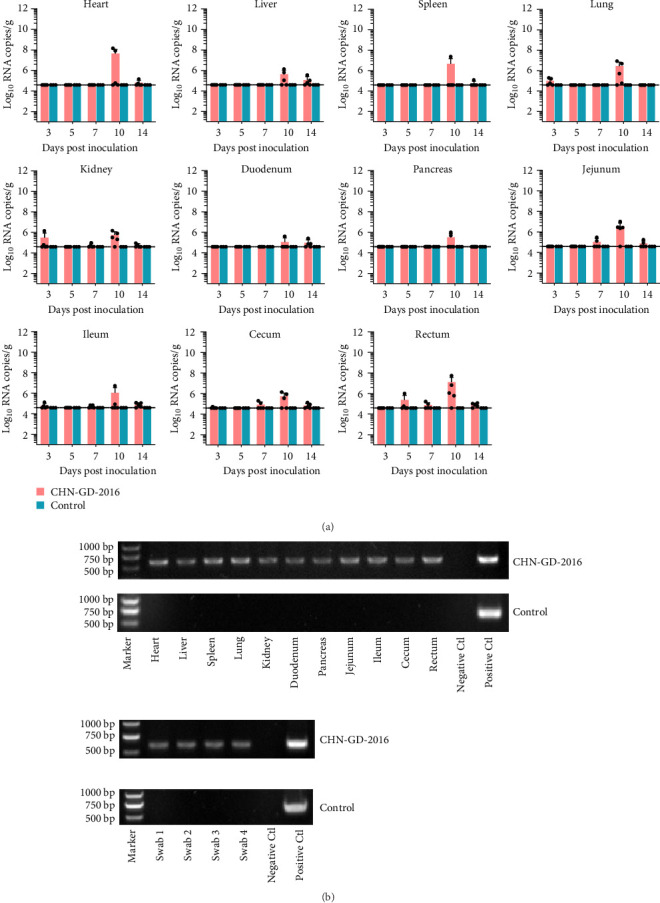
Distribution of virus RNA in heart, liver, spleen, lungs, kidneys, duodenum, pancreas, jejunum, ileum, cecum, and rectum of geese using qRT-PCR (A) and RT-PCR (B). The distribution and dynamics of PDCoV across different organs of inoculated and control geese were evaluated through qRT-PCR at 3, 5, 7, 10, and 14 dpi (A). The specific amplification of the PDCoV *S* gene fragment in tissue samples from inoculated group were tested positive by qRT-PCR at 7 dpi via RT-PCR (B). For rectal swab, swab1-4 were random selected from samples which were tested positive by qRT-PCR from 3, 4, 10 and 14 dpi, respectively. The detection limit for viral RNA in organ samples by qRT-PCR was determined to be 4.6 log_10_ copies/g of PDCoV, indicated by the dotted lines in the figure.

To investigate the dynamic distribution of PDCoV in duck and goose organs, viral RNA detection was conducted on organ samples collected at five specific time points: 3, 5, 7, 10, and 14 dpi, using qRT-PCR. Viral RNA copies were detected in all range of organs examined of PDCoV inoculated group (Figures 3A and 4A), indicating a systemic infection of PDCoV in ducks and geese.

The viral RNA copies in duck organs generally exhibited an increase-peak-decline trend. In parenchymal organs such as the heart, liver, spleen, lungs, and kidneys, the viral load was relatively low or undetectable at 3 and 5 dpi, but peaked at 7 dpi, displaying relatively high levels of viral load (ranging from 8.50 to 10.95 Log_10_ RNA copies/g), and subsequently decreased to lower levels by 14 dpi (Figure 3A). In the duodenum, the virus was detectable at 3 dpi (around 5.85 Log_10_ RNA copies/g), peaked at 5 dpi (around 8.00 Log_10_ RNA copies/g), and then gradually declined (Figure 3A). Unlike the sharp increase observed in the parenchymal organs, the viral loads in other digestive organs, including the pancreas, jejunum, ileum, cecum, and rectum, showed a steady increase (Figure 3A). Except for the jejunum, which peaked at 7 dpi (around 9.03 Log_10_ RNA copies/g), the viral loads in these organs peaked at 10 dpi (Figure 3A). The pancreas, jejunum, and cecum showed viral detection as early as 3 dpi, whereas the ileum and rectum did not (Figure 3A).

In organ samples collected from geese, the viral copies were found to peak at 10 dpi across all organs, with the heart displaying a relatively higher viral load (around 7.95 Log_10_ RNA copies/g) compared to other organs (ranging from 5.00 to 7.00 Log_10_ RNA copies/g; Figure 4A). The heart, liver, spleen, duodenum, and pancreas did not exhibit viral detection at 3, 5, and 7 dpi, while other organs showed viral detection at these time points, albeit at relatively low levels, slightly above the detection limit (Figure 3B). The overall viral load and number of positive geese samples are lower than those of the ducks, indicating weaker susceptibility of geese to PDCoV infection. To further test the presence of PDCoV genome copies, the qRT-PCR positive samples from 7 dpi were used to amplify *S* gene fragment using RT-PCR. As shown in Figures 3B and 4B, an expected 609-bp product was detected in these samples, these results confirmed the presence of PDCoV in the positive samples.

### 3.3. Pathological Lesions Occur in the Intestines of Inoculated Ducks

**Figure 5:**
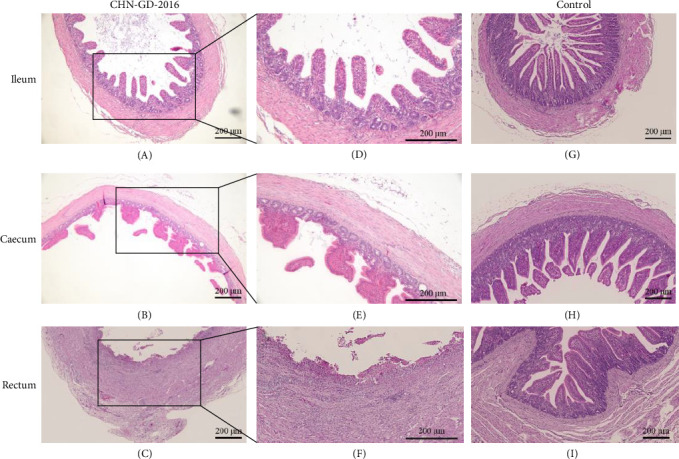
Pathology of tissue sections from ducks inoculated with PDCoV. Tissue sections from ducks and geese inoculated with PDCoV CHN-GD-2016 were collected at 7 dpi (A–F), along with tissues from the control group (G–I), including ileum (A, D, G), cecum (B, E, H), and rectum (C, F, I). After fixation, the tissues were stained for observation. D–F represent magnified views of A–C. Staining was performed using the hematoxylin and eosin (H&E) method.

Obvious tissue damage were observed in the intestines of inoculated ducks, indicating the severity of the pathological changes (Figure 5). Specifically, in the ileum region, a marked reduction in the quantity of villi was noted, accompanied by villus atrophy and shortening. These alterations suggest impaired nutrient absorption and disrupted intestinal function. Moving to the cecum, mucosal layer atrophy was evident, further highlighting the extensive impact on the intestinal lining. In the rectum, the damage was even more pronounced, with mucosal layer necrosis and shedding observed. Additionally, there was abundant heterophilic cell infiltration in the rectal tissue, indicating an active immune response to the infection. These findings collectively underscore the detrimental effects of the infection on the intestinal health of the ducks.

**Figure 6:**
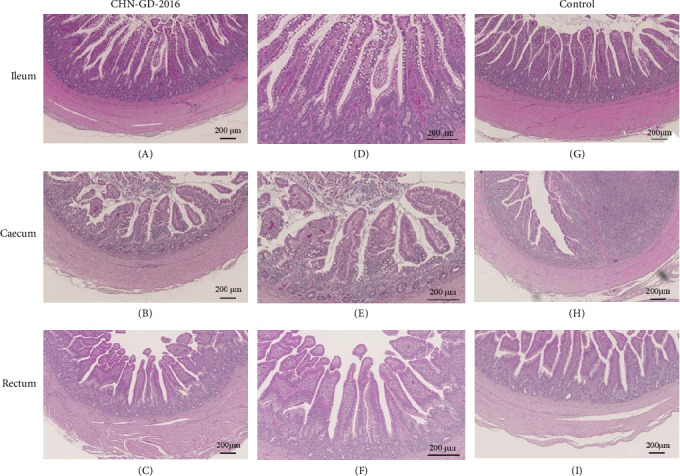
Pathology of tissue sections from geese inoculated with PDCoV. Tissue sections from ducks and geese inoculated with PDCoV CHN-GD-2016 were collected 10 dpi (A–F), along with tissues from the control group (G–I), including ileum (A, D, G), cecum (B, E, H), and rectum (C, F, I). After fixation, the tissues were stained for observation. D–F represent magnified views of A–C. Staining was performed using the hematoxylin and eosin (H&E) method.

In geese, histopathological examination of the intestinal tissues revealed no observable lesions (Figure 6). This result is consistent with the clinical signs and viral distribution, both of which indicated low levels of viral presence. These findings suggest that geese exhibit a degree of resistance or insensitivity to PDCoV infection. These results highlight the potential differences in susceptibility between ducks and geese to PDCoV.

### 3.4. Seroconversion Occurred in Inoculated Ducks and Geese

**Figure 7:**
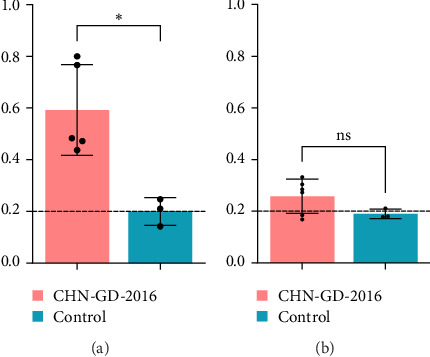
PDCoV-specific IgG antibody levels in duck serum samples (A) and goose serum samples (B) at 14 dpi. IgG antibody levels for PDCoV in duck (A) and goose (B) were evaluated using 14 dpi serum samples. The results are shown as the mean ± SD, with each point reflecting individual sample values. The dotted line marks the cut-off. ns, not significant; *p* < 0.05 (*⁣*^*∗*^).

The levels of IgG antibodies against PDCoV were evaluated using an indirect ELISA, as depicted in Figure 7. At 14 dpi, the inoculated group of ducks exhibited a notable elevation in IgG antibody levels, clearly indicating the presence of an antibody response against the virus. This increase in IgG antibodies suggests that the ducks' immune systems were actively responding to the PDCoV infection. In contrast, the IgG antibody levels in geese did not show a significant increase at the same time point. This observation suggests that the immune response of geese to PDCoV might differ from that of ducks, potentially indicating variations in susceptibility or immune mechanisms between these two avian species. Further research is needed to investigate these differences and their implications for PDCoV infection and immunity in geese.

### 3.5. No Genetic Mutations Were Observed in the *S* Gene Amplified From Samples of Inoculated Ducks and Geese Samples

To investigate whether genetic changes occurred in the *S* gene of PDCoV during experimental infection in animals, the complete *S* gene of the CHN-GD-2016 strain was amplified from both organ and swab samples collected from inoculated animals. Subsequently, these amplified *S* genes were sequenced. The sequenced *S* gene had a length of 3480 nucleotides, encoding a protein consisting of 1160 amino acids. Upon comparison with the CHN-GD-2016 strain that was cultured in cell lines, no mutations were observed in the *S* gene amplified from the animal samples. This finding suggests that the *S* gene of PDCoV remained stable during the experimental infection in animals and did not undergo genetic changes that could potentially affect its function or virulence (Supporting Information File S1).

## 4. Discussion

Coronaviruses, notorious for their broad host range and capacity to breach species barriers, pose a formidable threat to both human and animal health, as evidenced by the devastating global pandemics caused by SARS-CoV, MERS-CoV, and most recently, SARS-CoV-2. The potential for interspecies transmission underscores the risk of emerging novel infectious diseases.

Our study delved into the susceptibility of ducks and geese to PDCoV, revealing intriguing insights into its pathogenesis and transmission dynamics. In ducks, PDCoV triggered pronounced clinical manifestations, particularly watery diarrhea, which correlated closely with the viral RNA dynamics in rectal swabs. The virus exhibited a broad organ tropism, with distinct viral load patterns observed between parenchymal and digestive organs. Notably, the severity of clinical signs and viral shedding in ducks indicated a heightened adaptability of PDCoV to this host. Conversely, in geese, despite the detection of viral RNA in various organs, the absence of diarrhea and relatively low viral loads suggested a more muted response to PDCoV infection. Serological and histopathological analyses further corroborated these findings, revealing a robust antibody response and significant tissue damage in ducks, whereas geese exhibited minimal serological changes and limited tissue damage. The distribution of the virus in parenchymal organs and histopathological damage suggested possible viremia following PDCoV infection. Previous studies have shown the presence of viral RNA in tracheal swabs from PDCoV-inoculated and sentinel chickens during experiments [15], possibly related to ducks' lung damage. In this study, two peaks in viral RNA copies in duck feces and diarrhea scores were observed, with the peak of diarrhea scores occurring later than the peak of viral RNA copies in feces (Figure 2A,C). Additionally, the viral RNA copies in goose feces were consistently lower than those in duck feces at all time points (Figure 2C,D) and geese exhibited no diarrhea (Figure 2B). These findings suggest a strong correlation between diarrhea and viral RNA in feces in both ducks and geese, which may also reflect an increase in viral nucleic acid detection in internal organs (Figures 3 and 4). This observation is consistent with findings reported in other studies involving chickens [14, 15]. In pigs, PDCoV HKU15 has been detected in blood, liver, lungs, and kidneys, as well as in saliva and oral fluids [19–21].

Moreover, the potential involvement of multiple receptors in PDCoV infection, as suggested by recent research, adds another layer of complexity to its transmission dynamics. The exploration of PDCoV receptors, particularly in nonporcine hosts, is imperative to gain a deeper understanding of its pathogenesis and to develop targeted interventions. Beyond natural infections in wildlife, PDCoV has been proven to infect calves, chicks, turkeys, and chicken embryos by experimental infection. Given its broad host range, it is crucial to study PDCoV receptors. Aminopeptidase N (APN) is a transmembrane glycoprotein and a common receptor for HCoV-229E and TGEV [19, 21]. Two studies demonstrated that porcine APN (pAPN) serves as the entry receptor for PDCoV [20, 22]. Recent studies show that PDCoV's receptor-binding domain (RBD) can bind to human APN (hAPN) [23]. PDCoV can also infect pAPN-knockout pig intestinal epithelial (IPI-2I) cells, which suggests that PDCoV infection may involve other receptors [24]. Overall, PDCoV demonstrates strong interspecies infectivity.

The global distribution of PDCoV, coupled with its proven interspecies infectivity, underscores the urgency to unravel its transmission mechanisms. Our findings contribute to this endeavor by highlighting the differential susceptibility of ducks and geese to PDCoV. Epidemiological studies have shown widespread deltacoronaviruses infection in wild birds and domestic poultry [25], with reports of virus detection in China [26], the United States and Canada [13], Brazil [27], Australia [28], Finland [29], and the United Arab Emirates [30]. Our findings reveal contrasts in PDCoV susceptibility between ducks and geese. Ducks developed diarrhea coinciding with systemic viral dissemination and intestinal pathology, whereas geese showed no clinical signs and low-level viral RNA detection. Ducks developed seroconversion, whereas geese showed minimal antibody responses. These results implicate ducks as potential amplifiers of PDCoV transmission, while the role of geese in viral transmission requires further investigation to elucidate their epidemiological significance. Deltacoronaviruses infection rates are higher in aquatic wild birds than in terrestrial wild birds [14]. Ducks and geese have been proven to host other coronaviruses [31–33]. Therefore, as the most common domestic waterfowl and with close ties to pigs—a major agricultural animal intimately linked to human life—understanding the role of ducks and geese in PDCoV transmission is crucial for effective prevention and control strategies against coronaviruses.

## Data Availability

The data that support the findings of this study are available from the corresponding author upon reasonable request.

## Supporting Information

Supporting Information File S1: S Gene Sequences of PDCoV CHN-GD-2016 strain in Cell Culture and Duck's and Goose's Rectum Tissue.

## References

[1] Graham R. L., Baric R. S. (2010). Recombination, Reservoirs, and the Modular Spike: Mechanisms of Coronavirus Cross-Species Transmission. *Journal of Virology*.

[2] International Committee on Taxonomy of Viruses (2012). Family—Coronaviridae. *Virus Taxonomy: Ninth Report of the International Committee on Taxonomy of Viruses*.

[3] Dong B. Q., Liu W., Fan X. H. (2007). Detection of a Novel and Highly Divergent Coronavirus From Asian Leopard Cats and Chinese Ferret Badgers in Southern China. *Journal of Virology*.

[4] Woo P. C., Lau S. K., Lam C. S. (2009). Comparative Analysis of Complete Genome Sequences of Three Avian Coronaviruses Reveals a Novel Group 3c Coronavirus. *Journal of Virology*.

[5] Woo P. C., Lau S. K., Lam C. S. (2012). Discovery of Seven Novel Mammalian and Avian Coronaviruses in the Genus Deltacoronavirus Supports Bat Coronaviruses as the Gene Source of Alphacoronavirus and Betacoronavirus and Avian Coronaviruses as the Gene Source of Gammacoronavirus and Deltacoronavirus. *Journal of Virology*.

[6] Marthaler D., Raymond L., Jiang Y., Collins J., Rossow K., Rovira A. (2014). Rapid Detection, Complete Genome Sequencing, and Phylogenetic Analysis of Porcine Deltacoronavirus. *Emerging Infectious Diseases*.

[7] Ajayi T., Dara R., Misener M., Pasma T., Moser L., Poljak Z. (2018). Herd-Level Prevalence and Incidence of Porcine Epidemic Diarrhoea Virus (PEDV) and Porcine Deltacoronavirus (PDCoV) in Swine Herds in Ontario, Canada. *Transboundary and Emerging Diseases*.

[8] Song D., Zhou X., Peng Q. (2015). Newly Emerged Porcine *Deltacoronavirus* Associated With Diarrhoea in Swine in China: Identification, Prevalence and Full-Length Genome Sequence Analysis. *Transboundary and Emerging Diseases*.

[9] Jang G., Lee K.-K., Kim S.-H., Lee C. (2017). Prevalence, Complete Genome Sequencing and Phylogenetic Analysis of Porcine Deltacoronavirus in South Korea, 2014-2016. *Transboundary and Emerging Diseases*.

[10] Saeng-Chuto K., Lorsirigool A., Temeeyasen G. (2017). Different Lineage of Porcine Deltacoronavirus in Thailand, Vietnam and Lao PDR in 2015. *Transboundary and Emerging Diseases*.

[11] Lednicky J. A., Tagliamonte M. S., White S. K. (2021). Independent Infections of Porcine Deltacoronavirus Among Haitian Children. *Nature*.

[12] Kong F., Wang Q., Kenney S. P., Jung K., Vlasova A. N., Saif L. J. (2022). Porcine Deltacoronaviruses: Origin, Evolution, Cross-Species Transmission and Zoonotic Potential. *Pathogens*.

[13] Chen Q., Wang L., Yang C. (2018). The Emergence of Novel Sparrow Deltacoronaviruses in the United States More Closely Related to Porcine Deltacoronaviruses Than Sparrow Deltacoronavirus Hku17. *Emerging Microbes & Infections*.

[14] Boley P. A., Alhamo M. A., Lossie G. (2020). Porcine Deltacoronavirus Infection and Transmission in Poultry, United States. *Emerging Infectious Diseases*.

[15] Liang Q., Zhang H., Li B. (2019). Susceptibility of Chickens to Porcine Deltacoronavirus Infection. *Viruses*.

[16] Jung K., Hu H., Saif L. J. (2017). Calves Are Susceptible to Infection With the Newly Emerged Porcine Deltacoronavirus, but Not With the Swine Enteric Alphacoronavirus, Porcine Epidemic Diarrhea Virus. *Archives of Virology*.

[17] Xu Z., Zhong H., Zhou Q. (2018). A Highly Pathogenic Strain of Porcine Deltacoronavirus Caused Watery Diarrhea in Newborn Piglets. *Virologica Sinica*.

[18] Standardization Administration of the People’s Republic of China (2011). Laboratory Animal-Microbiological Standards and Monitoring.

[19] Delmas B., Gelfi J., L’Haridon R. (1992). Aminopeptidase N Is a Major Receptor for the Enteropathogenic Coronavirus TGEV. *Nature*.

[20] Li W., Hulswit R. J. G., Kenney S. P. (2018). Broad Receptor Engagement of An Emerging Global Coronavirus May Potentiate Its Diverse Cross-Species Transmissibility. *Proceedings of the National Academy of Sciences*.

[21] Yeager C. L., Ashmun R. A., Williams R. K. (1992). Human Aminopeptidase N Is a Receptor for Human Coronavirus 229E. *Nature*.

[22] Wang B., Liu Y., Ji C. M. (2018). Porcine Deltacoronavirus Engages the Transmissible Gastroenteritis Virus Functional Receptor Porcine Aminopeptidase N for Infectious Cellular Entry. *Journal of Virology*.

[23] Ji W., Peng Q., Fang X. (2022). Structures of a Deltacoronavirus Spike Protein Bound to Porcine and Human Receptors. *Nature Communications*.

[24] Zhu X., Liu S., Wang X. (2018). Contribution of Porcine Aminopeptidase N to Porcine Deltacoronavirus Infection. *Emerging Microbes & Infections*.

[25] Wille M., Holmes E. C. (2020). Wild Birds as Reservoirs for Diverse and Abundant Gamma- and Deltacoronaviruses. *FEMS Microbiology Reviews*.

[26] Chu D. K., Leung C. Y., Gilbert M. (2011). Avian Coronavirus in Wild Aquatic Birds. *Journal of Virology*.

[27] Torres C. A., Hora A. S., Tonietti P. O. (2016). Gammacoronavirus and Deltacoronavirus in Quail. *Avian Diseases*.

[28] Chamings A., Nelson T. M., Vibin J., Wille M., Klaassen M., Alexandersen S. (2018). Detection and Characterisation of Coronaviruses in Migratory and Non-Migratory Australian Wild Birds. *Scientific Reports*.

[29] Hepojoki S., Lindh E., Vapalahti O., Huovilainen A. (2017). Prevalence and Genetic Diversity of Coronaviruses in Wild Birds, Finland. *Infection Ecology & Epidemiology*.

[30] Lau S. K. P., Wong E. Y. M., Tsang C. C. (2018). Discovery and Sequence Analysis of Four Deltacoronaviruses From Birds in the Middle East Reveal Interspecies Jumping With Recombination as a Potential Mechanism for Avian-to-Avian and Avian-to-Mammalian Transmission. *Journal of Virology*.

[31] Chen G.-Q., Zhuang Q.-Y., Wang K.-C. (2013). Identification and Survey of a Novel Avian Coronavirus in Ducks. *PLoS ONE*.

[32] Zhuang Q.-Y., Wang K.-C., Liu S. (2015). Genomic Analysis and Surveillance of the Coronavirus Dominant in Ducks in China. *PLoS ONE*.

[33] Pauly M., Snoeck C. J., Phoutana V. (2019). Cross-Species Transmission of Poultry Pathogens in Backyard Farms: Ducks as Carriers of Chicken Viruses. *Avian Pathology*.

