# Supplementary material for: Differential Susceptibility to Porcine Deltacoronavirus: Ducks Show Greater Vulnerability Than Geese
Source: Transbound Emerg Dis. 2025 Jun 4;2025:2339024. doi: 10.1155/tbed/2339024 (PMC12158570; doi:10.1155/tbed/2339024)
Supplement: Supporting Information — File S1: S Gene Sequences of PDCoV CHN-GD-2016 strain in Cell Culture and Duck's and Goose's Rectum Tissue. [file 2339024.f1.pdf]

_S_ Gene Sequences of PDCoV CHN-GD-2016 strain in Cell Culture and Ferret Ileum Tissue.

Sequencing of all the amplified *S* gene samples produced consistent results. One duck (10 dpi) and one goose (10 dpi) in rectum tissue is shown here. In the duck and the goose samples, the *S* gene sequence matched that of CHN-GD-2016.

S gene sequence of CHN-GD-2016

ATGCAGAGAGCTCTATTGATTATGACCTTACTTTGTCTCGTTCGAGCAAAGTTTGCTGAT
GATCTACTCGATTTGCTCACCTTCCCGGGTGCACATCGCTTCTTACATAAACTCACGAGT
AATTCCAGCAGTCTCTACTCGCGGGCTAATAACTTTGATGTTGGCGTTCTTCCTGGCTA
CCCCACTAAGAACGTTAACCTCTTCTCACCACTTACTAACTCTACTTTGCCAATTAATGG
CCTTCATCGGAGTTACCAACCTCTTATGCTGAATTGTCTTACTAAAATAACTAACCACAC
TCTCAGCATGTATCTCCTACCTAGTGAGATACAAACTTATAGCTGCGGCGGTGCCATGG
TTAAATACCAGACACATGATGCAGTTCGTATCATTTTAGACCTCACTGTCACTGACCAC
ATCTCTGTTGAAGTCGTTGGCCAACGTGGTGAAAATTATGTGTTTGTTTGTAGTGAGCA
GTTTAACTATACCACTGCATTACCCAACTCTACCTTCTTCTCACTTAATTCTGAGCTTTAT
TGCTTTATTAATAACACCTACTTAGGTATTCTTCCACCTGATTTAACTGACTTTACGGTCT
ATCGTACTGGGCAGTTTTATGCTAATGGTTACCTTTTAGGTACTTTACCTATTACGGTTAA
CTATGTAAGGTTGTATCGGGGTCATTTGTCGGCCAATAGTGCCCACTTTGCCCTTGCAA
ACCTAACCGATACACTCATAACACTTACCAATACTACTATATCGCAAATCACTTATTGTG
ATAAGTCAGTAGTTGATTCAATAGCATGCCAGCGCTCTTCTCACGAAGTGGAGGATGGG
TTTTACTCCGACCCTAAATCTGCCGTTAGAGCTAGGCAACGTACTATTGTTACACTACCT
AAGCTCCCTGAGCTTGAAGTAGTGCAGTTAAATATTTCTGCACACATGGATTTTGGCGA
AGCCAGACTTGACAGCGTTACCATCAATGGTAACACATCCTATTGTGTCACTAAGCCTT
ACTTCAGGCTTGAAACTAACTTTATGTGTACAGGTTGCACTATGAATCTGCGCACTGAT
ACCTGTAGTTTTGACCTGTCAGCAGTAAACAATGGCATGTCATTCTCTCAATTCTGTCTA
AGCACTGAATCTGGTGCTTGTGAGATGAAAATTATTGTTACCTACGTATGGAATTACTTG
CTAAGGCAGCGTTTGTATGTTACAGCTGTAGAGGGCCAGACTCACACTGGAACCACTT
CAGTACATGCAACAGACACTTCTAGTGTAATCACTGATGTCTGCACTGATTACACTATCT
ATGGAGTCTCTGGTACTGGCATTATTAAGCCATCAGATCTCTTATTACATAATGGCATAG
CATTCACCTCTCCAACAGGTGAGCTCTATGCATTTAAAAATATAACCACTGGCAAAACC
CTTCAGGTCTTACCGTGTGAAACCCCTTCTCTACTGATTGTGATAAACAACACCGTTGT
CGGTGCTATCACATCCAGTAACTCAACTGAAAATAATAGGTTTACTACTACTATTGTCAC
ACCTACTTTCTTTTATTCCACAAATGCCACCACCTTCAACTGCACCAAGCCTGTTTTGT
CCTATGGACCCATCAGCGTGTGTAGTGATGGTGCAATTGCGGGAACATCCACATTACAG
AATACTCGACCATCCATAGTTTCACTATACGATGGCGAAGTTGAAATACCATCTGCATTT
TCTCTTTCTGTTCAGACGGAGTATTTGCAAGTTCAAGCAGAGCAAGTTATAGTTGATTG
TCCTCAGTATGTATGCAACGGCAACAGCCGTTGTCTACAATTACTGGCACAATACACCT
CAGCTTGCTCTAACATTGAAGCAGCTCTGCATTCCTCTGCACAGTTGGATAGCAGAGA
GATTATAAATATGTTTCAAACATCAACACAGTCCTTGCAGTTAGCTAATATTACCAACTT
CAAGGGTGACTACAATTTTAGCAGCATACTAACCACCAGACTAGGTGGCAGATCTGCTA
TTGAAGACCTTCTTTTTAATAAAGTTGTTACTAGTGGCCTTGGCACTGTTGATCAGGAC
TACAAAGCCTGCTCTAGAGACATGGCCATCGCTGACTTAGTTTGTTCCCAGTATTACAA
TGGCATCATGGTTCTACCTGGTGTTGTTGATGCTGAGAAAATGGCAATGTACACTGGCT
CTCTTACTGGAGCTATGGTATTTGGGGGACTGACTGCTGCAGCTGCAATACCCTTCGCT

ACAGCAGTACAAGCTCGCCTCAATTATGTCGCACTGCAAACAAATGTACTACAAGAAA
ACCAGAAAATTCTTGCAGAATCATTTAACCAAGCAGTTGGCAATATATCACTTGCACTA
TCTTCTGTTAATGATGCCATCCAGCAAACTTCTGAGGCTCTTAACACCGTAGCTATTGCT
ATTAAAAAGATTCAAACAGTTGTTAACCAGCAGGGTGAGGCATTATCACACCTGACTG
CACAGCTGTCAAACAATTTTCAAGCAATTTCGACTTCTATTCAAGACATTTACAACCGT
CTTGAGGAAGTAGAGGCTAACCAGCAAGTTGACCGTCTCATCACAGGACGGTTGGCT
GCACTTAATGCATATGTTACTCAGTTACTCAATCAGATGTCTCAGATTAGACAATCTCGA
TTGTTAGCTCAGCAAAAGATTAATGAGTGTGTCAAATCTCAGTCATCCAGATACGGTTT
CTGTGGAAATGGCACACACATCTTCTCACTTACACAGACTGCACCAAATGGCATATTTT
TCATGCATGCAGTGCTTGTACCCAACAAATTCACACGTGTCAACGCTTCTGCCGGCATT
TGTGTGGATAATATCAAAGGCTACTCATTGCAGCCTCAACTTATACTCTACCAGTTTAAT
AACTCCTGGAGAGTTACACCTAGAAATATGTATGAACCCAGACTGCCCCGGCAAGCTG
ATTTCATACAATTAACTGATTGCAGCGTTACTTTTTATAACACCACCGCTGCTAATCTTC
CCAATATTATCCCTGACATTATAGATGTCAATCAAACAGTCAGTGATATTATTGACAATTT
ACCTACAGCAACACCTCCTCAGTGGGATGTTGGTATCTATAACAACACTATTCTCAACC
TCACCGTTGAGATTAATGATCTACAAGAGCGGTCTAAAAACCTCTCACAGATTGCAGAT
CGTTTACAAAATTATATTGACAATCTTAACAATACTCTAGTTGACCTTGAATGGCTCAAC
AGAGTAGAAACTTACCTTAAATGGCCGTGGTATATATGGCTTGCCATTGCCCTGGCTCTT
ATTGCATTTGTGACAATCCTCATAACAATCTTTCTTTGTACTGGTTGTTGTGGTGGTTGC
TTTGGTTGTTGTGGCGGTTGTTTTGGCCTTTTCTCTAAGAAGAAAAGGTATACCGACGA
CCAACCAACACCGTCCTTTAAGTTTAAGGAATGGTAG

S gene sequence of one duck's rectum tissue from 10 dpi
ATGCAGAGAGCTCTATTGATTATGACCTTACTTTGTCTCGTTCGAGCAAAGTTTGCTGAT
GATCTACTCGATTTGCTCACCTTCCCGGGTGCACATCGCTTCTTACATAAACTCACGAGT
AATTCCAGCAGTCTCTACTCGCGGGCTAATAACTTTGATGTTGGCGTTCTTCCTGGCTA
CCCCACTAAGAACGTTAACCTCTTCTCACCACTTACTAACTCTACTTTGCCAATTAATGG
CCTTCATCGGAGTTACCAACCTCTTATGCTGAATTGTCTTACTAAAATAACTAACCACAC
TCTCAGCATGTATCTCCTACCTAGTGAGATACAAACTTATAGCTGCGGCGGTGCCATGG
TTAAATACCAGACACATGATGCAGTTCGTATCATTTTAGACCTCACTGTCACTGACCAC
ATCTCTGTTGAAGTCGTTGGCCAACGTGGTGAAAATTATGTGTTTGTTTGTAGTGAGCA
GTTTAACTATACCACTGCATTACCCAACTCTACCTTCTTCTCACTTAATTCTGAGCTTTAT
TGCTTTATTAATAACACCTACTTAGGTATTCTTCCACCTGATTTAACTGACTTTACGGTCT
ATCGTACTGGGCAGTTTTATGCTAATGGTTACCTTTTAGGTACTTTACCTATTACGGTTAA
CTATGTAAGGTTGTATCGGGGTCATTTGTCGGCCAATAGTGCCCACTTTGCCCTTGCAA
ACCTAACCGATACACTCATAACACTTACCAATACTACTATATCGCAAATCACTTATTGTG
ATAAGTCAGTAGTTGATTCAATAGCATGCCAGCGCTCTTCTCACGAAGTGGAGGATGGG
TTTTACTCCGACCCTAAATCTGCCGTTAGAGCTAGGCAACGTACTATTGTTACACTACCT
AAGCTCCCTGAGCTTGAAGTAGTGCAGTTAAATATTTCTGCACACATGGATTTTGGCGA
AGCCAGACTTGACAGCGTTACCATCAATGGTAACACATCCTATTGTGTCACTAAGCCTT
ACTTCAGGCTTGAAACTAACTTTATGTGTACAGGTTGCACTATGAATCTGCGCACTGAT
ACCTGTAGTTTTGACCTGTCAGCAGTAAACAATGGCATGTCATTCTCTCAATTCTGTCTA
AGCACTGAATCTGGTGCTTGTGAGATGAAAATTATTGTTACCTACGTATGGAATTACTTG
CTAAGGCAGCGTTTGTATGTTACAGCTGTAGAGGGCCAGACTCACACTGGAACCACTT

CAGTACATGCAACAGACACTTCTAGTGTAATCACTGATGTCTGCACTGATTACACTATCT
ATGGAGTCTCTGGTACTGGCATTATTAAGCCATCAGATCTCTTATTACATAATGGCATAG
CATTCACCTCTCCAACAGGTGAGCTCTATGCATTTAAAAATATAACCACTGGCAAAACC
CTTCAGGTCTTACCGTGTGAAACCCCTTCTCTACTGATTGTGATAAACAACACCGTTGT
CGGTGCTATCACATCCAGTAACTCAACTGAAAATAATAGGTTTACTACTACTATTGTCAC
ACCTACTTTCTTTTATTCCACAAATGCCACCACCTTCAACTGCACCAAGCCTGTTTTGT
CCTATGGACCCATCAGCGTGTGTAGTGATGGTGCAATTGCGGGAACATCCACATTACAG
AATACTCGACCATCCATAGTTTCACTATACGATGGCGAAGTTGAAATACCATCTGCATTT
TCTCTTTCTGTTCAGACGGAGTATTTGCAAGTTCAAGCAGAGCAAGTTATAGTTGATTG
TCCTCAGTATGTATGCAACGGCAACAGCCGTTGTCTACAATTACTGGCACAATACACCT
CAGCTTGCTCTAACATTGAAGCAGCTCTGCATTCCTCTGCACAGTTGGATAGCAGAGA
GATTATAAATATGTTTCAAACATCAACACAGTCCTTGCAGTTAGCTAATATTACCAACTT
CAAGGGTGACTACAATTTTAGCAGCATACTAACCACCAGACTAGGTGGCAGATCTGCTA
TTGAAGACCTTCTTTTTAATAAAGTTGTTACTAGTGGCCTTGGCACTGTTGATCAGGAC
TACAAAGCCTGCTCTAGAGACATGGCCATCGCTGACTTAGTTTGTTCCCAGTATTACAA
TGGCATCATGGTTCTACCTGGTGTTGTTGATGCTGAGAAAATGGCAATGTACACTGGCT
CTCTTACTGGAGCTATGGTATTTGGGGGACTGACTGCTGCAGCTGCAATACCCTTCGCT
ACAGCAGTACAAGCTCGCCTCAATTATGTCGCACTGCAAACAAATGTACTACAAGAAA
ACCAGAAAATTCTTGCAGAATCATTTAACCAAGCAGTTGGCAATATATCACTTGCACTA
TCTTCTGTTAATGATGCCATCCAGCAAACTTCTGAGGCTCTTAACACCGTAGCTATTGCT
ATTAAAAAGATTCAAACAGTTGTTAACCAGCAGGGTGAGGCATTATCACACCTGACTG
CACAGCTGTCAAACAATTTTCAAGCAATTTCGACTTCTATTCAAGACATTTACAACCGT
CTTGAGGAAGTAGAGGCTAACCAGCAAGTTGACCGTCTCATCACAGGACGGTTGGCT
GCACTTAATGCATATGTTACTCAGTTACTCAATCAGATGTCTCAGATTAGACAATCTCGA
TTGTTAGCTCAGCAAAAGATTAATGAGTGTGTCAAATCTCAGTCATCCAGATACGGTTT
CTGTGGAAATGGCACACACATCTTCTCACTTACACAGACTGCACCAAATGGCATATTTT
TCATGCATGCAGTGCTTGTACCCAACAAATTCACACGTGTCAACGCTTCTGCCGGCATT
TGTGTGGATAATATCAAAGGCTACTCATTGCAGCCTCAACTTATACTCTACCAGTTTAAT
AACTCCTGGAGAGTTACACCTAGAAATATGTATGAACCCAGACTGCCCCGGCAAGCTG
ATTTCATACAATTAACTGATTGCAGCGTTACTTTTTATAACACCACCGCTGCTAATCTTC
CCAATATTATCCCTGACATTATAGATGTCAATCAAACAGTCAGTGATATTATTGACAATTT
ACCTACAGCAACACCTCCTCAGTGGGATGTTGGTATCTATAACAACACTATTCTCAACC
TCACCGTTGAGATTAATGATCTACAAGAGCGGTCTAAAAACCTCTCACAGATTGCAGAT
CGTTTACAAAATTATATTGACAATCTTAACAATACTCTAGTTGACCTTGAATGGCTCAAC
AGAGTAGAAACTTACCTTAAATGGCCGTGGTATATATGGCTTGCCATTGCCCTGGCTCTT
ATTGCATTTGTGACAATCCTCATAACAATCTTTCTTTGTACTGGTTGTTGTGGTGGTTGC
TTTGGTTGTTGTGGCGGTTGTTTTGGCCTTTTCTCTAAGAAGAAAAGGTATACCGACGA
CCAACCAACACCGTCCTTTAAGTTTAAGGAATGGTAG

S gene sequence of one goose’s rectum tissue from 10 dpi
ATGCAGAGAGCTCTATTGATTATGACCTTACTTTGTCTCGTTCGAGCAAAGTTTGCTGAT
GATCTACTCGATTTGCTCACCTTCCCGGGTGCACATCGCTTCTTACATAAACTCACGAGT
AATTCCAGCAGTCTCTACTCGCGGGCTAATAACTTTGATGTTGGCGTTCTTCCTGGCTA
CCCCACTAAGAACGTTAACCTCTTCTCACCACTTACTAACTCTACTTTGCCAATTAATGG

CCTTCATCGGAGTTACCAACCTCTTATGCTGAATTGTCTTACTAAAATAACTAACCACAC
TCTCAGCATGTATCTCCTACCTAGTGAGATACAAACTTATAGCTGCGGCGGTGCCATGG
TTAAATACCAGACACATGATGCAGTTCGTATCATTTTAGACCTCACTGTCACTGACCAC
ATCTCTGTTGAAGTCGTTGGCCAACGTGGTGAAAATTATGTGTTTGTTTGTAGTGAGCA
GTTTAACTATACCACTGCATTACCCAACTCTACCTTCTTCTCACTTAATTCTGAGCTTTAT
TGCTTTATTAATAACACCTACTTAGGTATTCTTCCACCTGATTTAACTGACTTTACGGTCT
ATCGTACTGGGCAGTTTTATGCTAATGGTTACCTTTTAGGTACTTTACCTATTACGGTTAA
CTATGTAAGGTTGTATCGGGGTCATTTGTCGGCCAATAGTGCCCACTTTGCCCTTGCAA
ACCTAACCGATACACTCATAACACTTACCAATACTACTATATCGCAAATCACTTATTGTG
ATAAGTCAGTAGTTGATTCAATAGCATGCCAGCGCTCTTCTCACGAAGTGGAGGATGGG
TTTTACTCCGACCCTAAATCTGCCGTTAGAGCTAGGCAACGTACTATTGTTACACTACCT
AAGCTCCCTGAGCTTGAAGTAGTGCAGTTAAATATTTCTGCACACATGGATTTTGGCGA
AGCCAGACTTGACAGCGTTACCATCAATGGTAACACATCCTATTGTGTCACTAAGCCTT
ACTTCAGGCTTGAAACTAACTTTATGTGTACAGGTTGCACTATGAATCTGCGCACTGAT
ACCTGTAGTTTTGACCTGTCAGCAGTAAACAATGGCATGTCATTCTCTCAATTCTGTCTA
AGCACTGAATCTGGTGCTTGTGAGATGAAAATTATTGTTACCTACGTATGGAATTACTTG
CTAAGGCAGCGTTTGTATGTTACAGCTGTAGAGGGCCAGACTCACACTGGAACCACTT
CAGTACATGCAACAGACACTTCTAGTGTAATCACTGATGTCTGCACTGATTACACTATCT
ATGGAGTCTCTGGTACTGGCATTATTAAGCCATCAGATCTCTTATTACATAATGGCATAG
CATTCACCTCTCCAACAGGTGAGCTCTATGCATTTAAAAATATAACCACTGGCAAAACC
CTTCAGGTCTTACCGTGTGAAACCCCTTCTCTACTGATTGTGATAAACAACACCGTTGT
CGGTGCTATCACATCCAGTAACTCAACTGAAAATAATAGGTTTACTACTACTATTGTCAC
ACCTACTTTCTTTTATTCCACAAATGCCACCACCTTCAACTGCACCAAGCCTGTTTTGT
CCTATGGACCCATCAGCGTGTGTAGTGATGGTGCAATTGCGGGAACATCCACATTACAG
AATACTCGACCATCCATAGTTTCACTATACGATGGCGAAGTTGAAATACCATCTGCATTT
TCTCTTTCTGTTCAGACGGAGTATTTGCAAGTTCAAGCAGAGCAAGTTATAGTTGATTG
TCCTCAGTATGTATGCAACGGCAACAGCCGTTGTCTACAATTACTGGCACAATACACCT
CAGCTTGCTCTAACATTGAAGCAGCTCTGCATTCCTCTGCACAGTTGGATAGCAGAGA
GATTATAAATATGTTTCAAACATCAACACAGTCCTTGCAGTTAGCTAATATTACCAACTT
CAAGGGTGACTACAATTTTAGCAGCATACTAACCACCAGACTAGGTGGCAGATCTGCTA
TTGAAGACCTTCTTTTTAATAAAGTTGTTACTAGTGGCCTTGGCACTGTTGATCAGGAC
TACAAAGCCTGCTCTAGAGACATGGCCATCGCTGACTTAGTTTGTTCCCAGTATTACAA
TGGCATCATGGTTCTACCTGGTGTTGTTGATGCTGAGAAAATGGCAATGTACACTGGCT
CTCTTACTGGAGCTATGGTATTTGGGGGACTGACTGCTGCAGCTGCAATACCCTTCGCT
ACAGCAGTACAAGCTCGCCTCAATTATGTCGCACTGCAAACAAATGTACTACAAGAAA
ACCAGAAAATTCTTGCAGAATCATTTAACCAAGCAGTTGGCAATATATCACTTGCACTA
TCTTCTGTTAATGATGCCATCCAGCAAACTTCTGAGGCTCTTAACACCGTAGCTATTGCT
ATTAAAAAGATTCAAACAGTTGTTAACCAGCAGGGTGAGGCATTATCACACCTGACTG
CACAGCTGTCAAACAATTTTCAAGCAATTTCGACTTCTATTCAAGACATTTACAACCGT
CTTGAGGAAGTAGAGGCTAACCAGCAAGTTGACCGTCTCATCACAGGACGGTTGGCT
GCACTTAATGCATATGTTACTCAGTTACTCAATCAGATGTCTCAGATTAGACAATCTCGA
TTGTTAGCTCAGCAAAAGATTAATGAGTGTGTCAAATCTCAGTCATCCAGATACGGTTT
CTGTGGAAATGGCACACACATCTTCTCACTTACACAGACTGCACCAAATGGCATATTTT
TCATGCATGCAGTGCTTGTACCCAACAAATTCACACGTGTCAACGCTTCTGCCGGCATT

TGTGTGGATAATATCAAAGGCTACTCATTGCAGCCTCAACTTATACTCTACCAGTTTAAT
AACTCCTGGAGAGTTACACCTAGAAATATGTATGAACCCAGACTGCCCCGGCAAGCTG
ATTTCATACAATTAACTGATTGCAGCGTTACTTTTTATAACACCACCGCTGCTAATCTTC
CCAATATTATCCCTGACATTATAGATGTCAATCAAACAGTCAGTGATATTATTGACAATTT
ACCTACAGCAACACCTCCTCAGTGGGATGTTGGTATCTATAACAACACTATTCTCAACC
TCACCGTTGAGATTAATGATCTACAAGAGCGGTCTAAAAACCTCTCACAGATTGCAGAT
CGTTTACAAAATTATATTGACAATCTTAACAATACTCTAGTTGACCTTGAATGGCTCAAC
AGAGTAGAAACTTACCTTAAATGGCCGTGGTATATATGGCTTGCCATTGCCCTGGCTCTT
ATTGCATTTGTGACAATCCTCATAACAATCTTTCTTTGTACTGGTTGTTGTGGTGGTTGC
TTTGGTTGTTGTGGCGGTTGTTTTGGCCTTTTCTCTAAGAAGAAAAGGTATACCGACGA
CCAACCAACACCGTCCTTTAAGTTTAAGGAATGGTAG
